# Southern Medical College Commencement

**Published:** 1881-03-20

**Authors:** 


### EDITORIALS AND MISCELLANEOUS.

### *SOUTHERN MEDICAL COLLEGE COMMENCEMENT.*

The Commencement exercises of the Southern Medical College took place at DeGive's Opera House, Atlanta, on Thursday evening, March 3d, 1881.

Notwithstanding the inclement weather a very large audience was present, composed of intelligent and refined citizens—the *elite* of the city. The occasion was made the more attractive by the presence of numerous ladies, and the intervals of the various parts of the programme were enlivened with strains of delightful music.

The exercises were opened with prayer by the Rev. Mr. Spalding. After which the Diplomas were delivered to thirty-eight graduates. They were arranged in two classes upon the stage. The members were separately served, each advancing as his name was called, and bowing politely, received his Diploma at the hands of Dr. Word, the Dean. When the entire class had thus received their Diplomas, they stood erect, at the sound of the gavel, and had the degree of Doctor of Medicine conferred by Dr. T. S. Powell, the President. The second class then received their Diplomas in like manner, the degree being conferred in Latin; then, at the sound of the gavel, both classes stood erect and were addressed by President Powell, in the following language:

*My Young Brethren:*

According to the laws of Georgia and the usages of the medical profession in America, you are now Doctors of Medicine. But you should remember that the work of instruction is not complete; its cares, its toils, its longings, its pleasures, are still before you.

If you wish to reach the apex of fame's pyramid you must enter upon your new duties from this moment, like zealous physicians just setting forth on a morning of promise, determined to bear aloft the proud flag of your profession, to advance as far as possible, the cause of humanity. May the world receive you kindly. May you be greeted with the kind words of a host of friends.

If you should not, be not discouraged, but pursue your noble employment with cheerfulness, and strive with increased ardor to enlarge its boundaries and contemplate the power with gratitude to God, which he has enabled you to acquire over the suffering and wretchedness of your fellow men, keeping steadily in view the great responsibilities which rest upon you.

Be true to the cause of science, to the dignity of your profession, to the reputation of your Alma Mater. Emulate the great and the good in all of your aspirations. Let all of the influence which you may be able to exercise in society be exerted for purposes which are dear to the patriot, the philanthropist and the Christian. Do this, and you will feel—indeed, you will know, whether life for you be robed in sunlight or darkened by trouble and tempest. Glory's temple will be the tomb, and death itself be immortality.

Go, now, my young brethren, and our prayers shall ever be, that when the trumpet-notes of the resurrection are sounded, and the muster-roll of the universe is called, that each one of you may rise from your mounds, and with the shout of divine victory on your lips, join the immortal legions of the skies.

The Ad Eundem degree was then conferred upon Dr. A F F Kerstan, an intelligent German physician of the city, and a graduate of the Kentucky School of Medicine. While the Diplomas were being delivered, the plaudits of the audience were frequent—numerous floral offerings were showered upon the stage, and rich boquets and vases laden with gorgeous and beautiful flowers were sent up to many of the class.

The Annual Address, by the Rev. C. A. Evans, next followed. We have not space for its insertion here, but it was, in all respects, an able and admirable production, and was listened to with profound interest and attention.

The Valedictory, by Dr. J. H. Redding, a member of the class, was excellent, and evinced much study and careful preparation. It was well delivered, and listened to with marked attention.

Prof. John Thad Johnson then conferred a prize (a Surgical Case) upon Dr. J. H. Redding for the best examination on the "Theory and Practice of Surgery." The professor stated that the examinations were thorough and the contest close with a large number. Honorable mention was made of Drs. G. A. Vinson, F. G. Donehoo and N. O. Harris.

A gold medal was offered by Prof. Jas. A. Gray for the best examination in "Chemistry, Surgery and Diseases of the Eye and Ear." This also was awarded to Dr. J. H. Redding.

Profs. Nicolson and Gray awarded a Medical Case to Mr. W. D. Vinson for the best examination as a first course student, in all the branches.

The names of the graduates are as follows:

| | | |
|---|---|---|
| W. B. ALLGOOD, | J. M. BEARDEN, | J. A. CANNON. |
| S. P. CLAYTON, | W. E. COURSON, | F. G. DONEHOO. |
| W. A. EILAND, | H. T. EMERSON, | R. L. FITE. |
| W. M. GAY, | C. C. GREEN, | J. H. GREEN. |
| A. GULLETT, | N. O. HARRIS, | M. A. HAYES, |
| T. F. HOUSTON, | H. L. HOWARD, | A. S JOHNSON. |
| C. A. JORDAN, | F. A. LIDDELL, | M. L. MAHAFFEY. |
| R. F. McCONNELL, | R. J. MILAM, | M. D. MILES. |
| W. B. PARKS, | F. H. PECK, | J. H. REDDING. |
| W. C. ROBINSON, | J. A. SISK, | J. R. STODGHILL. |
| F. G. THOMASON, | J. C. TRENTHAM, | T. L. TURK. |
| G. A. VINSON, | W. P. WALKER, | J. R. WEBB. |
| F. B. WRIGHT, | G. W. WRIGHT. | |

At the conclusion of the programme Dr. R. C. Word, the Dean, addressed the audience briefly, as follows:

We have now to state that the exercises of the evening are over. The number of the graduating class has perhaps surprised many. It is certainly a large class for a new Institution, and would have been considerably larger had not the prevalence of the mumps and measles prevented a number from completing their studies. In respect to morals, intelligence aud proficiency in their studies, the present class attained to a very high average.

I will add that the Faculty and Board of Trustees are much gratified at the presence, despite the inclement evening, of this large and intelligent audience.

They regard it as indicative of sympathy and interest in the great educational work in which we are engaged, and as furnishing another instance of the high public spirit of our people, and the generous encouragement which they have ever extended to all enterprises looking to the interest of the city and to the public good. Herein is found one of the secrets of the unprecedented growth and prosperity of Atlanta.

In the present instance both the public press and the citizens generally, have warmly co-operated in the efforts of the founders to establish the enterprise, which now is regarded as an assured success. We have already accomplished much, but have yet more in view.

A hospital must be erected in connection with the Institution before the next session, and other important facilities added. We aim at nothing less than to make it the great, leading, central Institution in the Southern States. One, of which the city will be proud, and in which the Profession throughout the South will rejoice. One, which in years to come it will be considered a high honor to have attended, and whose diploma will be a talisman and passport into the highest professional circles in the land. We thank the audience for their presence and polite attention.

The benediction was then pronounced by the Rev. A. G. Thomas, and the audience retired pleased with the occasion, and deeply impressed with the remarkable success of the new Institution and the great prospective advantages which it gives to medical education in the South.

